# Germ Origin of Disease as Seen by a Naturalist

**Published:** 1884-06-20

**Authors:** W. S. Newlon

**Affiliations:** Oswego, Kansas


### GERM ORIGIN OF DISEASE AS SEEN BY A NATURALIST.

By W. S. Newlon, M. D., Oswego, Kansas.

There are other ways to determine the germ origin of disease besides the use of the microscope. If confined to this instrument there are many scientists and physicians on account of defective sight and other causes that would never be able to make investigations in this interesting field or draw any correct inferences relating to it. Recently by the aid of the glass, however, rich discoveries have been made in microscopic germs causing disease, and we are to expect in the near future these discoveries will be enlarged and crystalized into usefulness and others, grand and noble will be added to them. We are in danger at the same time of making this a rage and a pet, of imputing too many of our maladies to these mysterious agents. We should when possible wait for positive facts in all mysterious matters of science before forming an opinion. But we cannot depend on the microscope in all diseases for facts.

Some germs causing disease are so small that nothing but the Divine Mind that formed them can see and estimate them.

The power of the microscope in the near future may be so increased that we can look deeper and further into the microscopic world. Beyond its vision, however, there will be noxious germs, no doubt, living, moving and having life no glass ever invented by humanity will reveal to us. How are we to suspect that such creatures, zoological and floral, do exist? Can we not say to almost a certainty they do exist in some cases by reasoning? If so, how?

When a disease has a germ origin it presents to us in its course some of the traits or phenomena of organic life, animal or floral.

Let us name a few of those phenomena and then apply them to our common diseases:

1st. Animals and plants are so nearly similar that what fact will apply to one applies to the other also. An egg of an animal and a seed of a plant are similar structures and the beginning of all organic life. After life is originated in many cases it may be propagated by buds. To start life from seeds and eggs they must be submitted to either germination or incubation and consequent fermentation.

2nd. Germination or incubation puts in motion a force called fermentation or zymosis. No organic body can decay and rot without fermentation. Flesh, fruit and all organic bodies would never decay if millions of germs did not incubate in every fibre and atom and set up fermentation and consequent decay.

The tooth of oxygen may eat iron and nearly all metals without germ life, but when organic bodies decay incubation or germination starts the process. In some countries meat is elevated in the air above germ life and this saves it from putrefaction even when the days are hot. Fruit is sealed in cans not to keep out air but germ life. In any disease then where there is putrefaction and fermentation or zymosis there is abundant germ life for a cause.

3rd. There are floral and zoological zones around the earth running with the equator or parallels, and each zone has its peculiar flora and faun. If the elephant and other phenomenal animals and plants live in such zones, microscopic animals and plants causing disease will do also.

4th. Some plants and animals flourish in certain regions—in swamps, in valleys dark and cold—and some are marine or aquatic and some inhabit sandy plains. When germs of this kind cause disease we call the malady endemic.

5th. Animals can travel with the wind and against it. Plants are carried by streams and the wind and by animals, and never against the wind.

6th. Plants grow in the warm days of summer, rest in winter and with the spring revive again. Insects also live in the summer.

7th. Animals may be entozoal and live in the dark, true parasites, but plants cannot flourish in the dark. Wood and Gray both say plants must have light for healthy growth. When plants cause disease we must suppose they grow on the surface of the body in the light. Animal germs may live anywhere in the body in the dark and in the light.

8th. Animals and plants are not attacked by parasites when in perfect health like when they are sickly and weak.

9th. Certain agents destroy organic life when applied to it. We call these agents antiseptics.

Let us now try some of our common diseases by these traits or forms of organic life and see whether we can find any similarity.

Typhus and typhoid fevers, have plantings and growings. Zymotic, zoological zone for former. Antiseptics influence them.

Small-pox, scarlatina, measles and other eruptions—planting and hatching, travel against the wind, zymotic zones for some of them. Antiseptics influence.

Whooping cough and mumps—planted and incubation, travel against the wind. Spread by germs.

Ague and intermittent fever—planted and germinate. Travel from swamps with wind, flourish in the summer, rest in the winter, start up to life in the spring. Antiseptics influence.

Goitre—endermic to mountain valleys; affect the weak; is influenced by antiseptics.

Consumption and scrofula—Seem to grow after planting; afflict the weak; have a zone; affected by antiseptics; grow in the dark; seem to be animal germs causing them.

Cancer and lupus—incubated and grow to maturity; may be planted; seems to spread by pudding like protozans.

Gonorrhœa and syphilis—planted seem to incubate and at maturity bear seeds or eggs; affected by antiseptics.

Leprosy and skin diseases—grow in the light; afflict the weak; spread to planting.

Hydrophobia, snake bite and skunk bite—grow by planting; seem to be affected by antiseptics; zymotic.

Rheumatism—flourishes in damp weather; influenced by antiseptics. Said to be zymotic.

Yellow fever—has a zone; flourishes in warm weather like the plants and hexapods.

Erysipelas, influenza, puerpural fever, typhoid pneumonia and putrid sore throat—spread by planting; affected by antiseptics; zymotic.

The above observations were made in a great hurry in a busy life and are not claimed to be perfect by any means, but they open a new field for thought and study so far as I know. This is my apology.—*Peoria Med. Monthly.*

